# Production of Ribosomal Protein S12/Renilla Luciferase Fusion and Development of a Bioluminescent Method for Detection of Aminoglycosides in Pork and Studying Its Recognition Mechanism

**DOI:** 10.3390/foods12020284

**Published:** 2023-01-07

**Authors:** Wanqiu Xia, Lei Zhang, Jianping Wang

**Affiliations:** 1 College of Veterinary Medicine, Hebei Agricultural University, Baoding 071000, China; 2 Veterinary Biological Technology Innovation Center of Hebei Province, Baoding 071000, China

**Keywords:** aminoglycosides, receptor, intermolecular interaction mechanism, ribosomal protein S12/luciferase fusion, bioluminescent method, pork

## Abstract

In this study, the genes of *Escherichia coli* ribosomal protein S12 and renilla luciferase were linked and expressed to produce a fusion protein, and its intermolecular interactions and affinities with sevenaminoglycosides were studied. Then, the fusion protein was used as the core agent to develop a bioluminescent method on a conventional microplate for determination of the residues of thesevenaminoglycosides in pork. This method contained only one sample-loading step, and thus the assay was finished within 30 min. The limits of detection for the sevendrugs were in the range of 0.51–1.1 ng/mL, and the sensitivity for a specific drug was mainly determined by the receptordrug affinity but not related with the binding energy. After general comparison, the present method showed generally better performances than the previously reported enzyme-linked immunosorbent assays for aminoglycosides. This is the first study reporting the recognition mechanisms of *Escherichia coli* ribosomal protein S12 for aminoglycosides and developing a bioluminescent method for detection of aminoglycoside residues in pork samples.

## 1. Introduction

**Figure 1:**
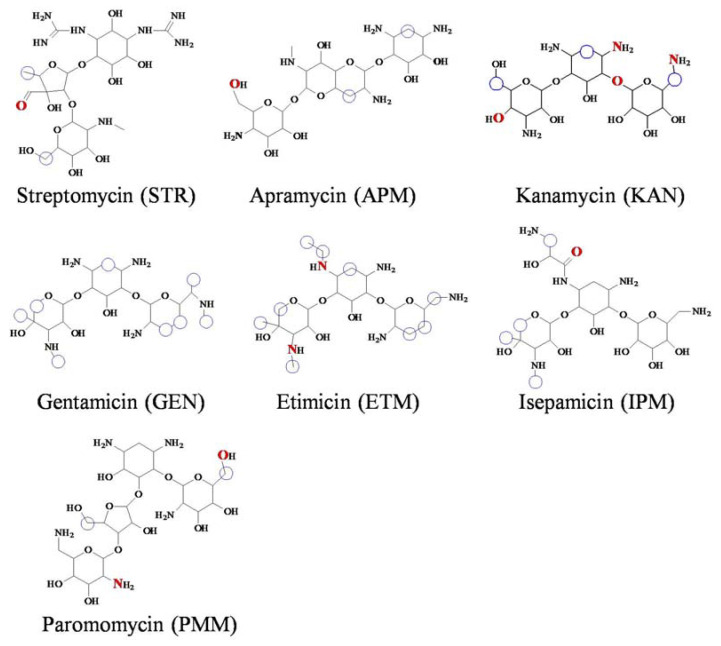
Molecular structures of the 7AGs (the atomsof red color interacted with the receptor via a hydrogen bond; the atoms circled with blue rings interacted with the receptor via hydrophobic interaction).

Aminoglycoside drugs (AGs, Figure 1) are usually used to treat the bacterial infections in animals, and their wide uses in food-producing animals will cause their residues in the foods of animal origin. However, AGs have severe ototoxicity and nephrotoxicity [1], and thusthe inspection of their residues in animal-derived foods are very important. For the protection of consumer health, the People’s Republic of China has set strict maximum residue limits for AGs in meat, e.g., streptomycin, 600 μg/kg; gentamicin and kanamycin, 100 μg/kg [2].

Up untilnow, different analytical methods have been developed to determinethe residues of AGs in food samples [3,4], and immunoassay is the most commonly used method for the analysis of a large number of samples. During the past two decades, enzyme-linked immunosorbent assay [5,6,7,8,9,10], fluorometric immunoassay [11], fluorescence polarization immunoassay [12,13], chemiluminescent immunoassay [14], and immunochromatographic strip [15,16,17] have been reported to determine AGs residues.

For an immunoassay, the key material is the used recognition reagent. All of the above-mentioned immunoassays for AGs use polyclonal antibody or monoclonal antibody as the recognition reagent. As shown in Figure 1, the molecular structures of commonly used AGs are different, and thereforethe antibody derivedfrom one specific drug shows different recognition abilities for other analogs. As a result, most of the previously reported immunoassays can only determine one drug [5,7,8,10,11,12,13,14,15,16,17], and only two immunoassays can determine two AGs [6,9]. This is because an antibody capable of recognizing more AGs species is not available. Thus, the finding of a recognition reagent showinga broad recognition spectrum is very necessary.

Compared with antibodies, receptorsare natural macromolecules that can recognize all of theirligands. Thus, the specific receptor of one class of drugs can be used as the broad specific recognition reagent to develop the multi-analytes analytical method. By now, there have been some papers reporting the development of receptor-based pseudo immunoassays for detection of different classes of analytes [18,19]. For example, a natural dihydropteroate synthase (the receptor of sulfonamides) wasproduced in our recent report, and a pseudo immunoassay wasdeveloped for the determination of 40 sulfonamides [20]. The results show that the detection spectrum wasbroader than all of the previously reported immunoassays for sulfonamides.

It is well known that the active site of AGs is the ribosomal protein S12 (RpsL12) of the prokaryotic ribosome, and thusRpsL12 is the receptor of AGs [21,22,23]. Furthermore, some reports have shown that the mutations of RpsL12 can lead to AG-resistant bacterial strains [24,25]. Therefore, it can be used as the broad specific recognition reagent to develop a multi-analyte analytical method for AGs. In our recent report, the RpsL12 of *Lysinibacillussphaericus* was produced, and a fluorescence polarization assay based on this receptor wasdeveloped in order to determine AG residues in pork [26]. The results showed that the detection spectrum wasbroader than all of the antibody-based immunoassays for AGs.

It is well known that the signal of an immunoassay is usually based on absorbancy, fluorescenceintensity, or luminescence intensity. Among the three signal systems, the luminescent signal is the most sensitive. Therefore, the chemiluminescence immunoassay has been used for the analysis of many analytes [27]. In comparison with the chemiluminescence method, bioluminescent methods are usually based on different photoproteins, e.g., firefly luciferase and renilla luciferase [28]. These enzymes can catalyze the specific substrate to emit light, and thusthey are widely used as the reporter proteins in the field oflifesciences. During the past few years, some researchers have produced luciferase-fused antibodies and developed some bioluminescent immunoassays for the detection of bacteria [29], proteins [30,31], and small molecule substances [32,33,34,35]. These reports show that the operation steps of the bioluminescent immunoassay are fewer than that of the chemiluminescence immunoassay, the assay time of the bioluminescent immunoassay is shorter than that of the chemiluminescence immunoassay, and the required substrate reagents of the bioluminescent immunoassay are fewer than that of the chemiluminescence immunoassay. As far as we know, however, a bioluminescent immunoassay capable of determining AGs residues has not been reported.

As discussed above, the recognition spectrum of *Lysinibacillussphaericus* RpsL12 for AGs is broader than that of the conventional antibodies, but whetherthe RpsL12 proteinsof other bacterial strainsalso show a broad recognition spectrum for AGs is unknown, and their recognition mechanisms for AGs are also unknown. *Escherichia coli* is the most frequently identified foodborne pathogen, and AGs are the most commonly used drugsto treat *Escherichia coli*-induced infections in food-producing animals. Therefore, the genes of *Escherichia coli* RpsL12 and renilla luciferase (Rluc) were linked and expressed to produce a novel fusion protein RpsL12–Rlucin the present study. Its intermolecular interactions and affinities with sevenAGs were tested. Then, the fusion protein was used to develop an indirect competitive bioluminescent method for the determination of AG residues in pork.

## 2. Materials and Methods

### 2.1. Reagents

The standard of etimicin (ETM) was the product of the China National Institute for Food and Drug Control (Beijing, China). The standards of gentamicin (GEN), kanamycin (KAN), streptomycin (STR), paromomycin (PMM), apramycin (APM), isepamicin (IPM), and bovine serum albumin (BSA) were the products of Shanghai Yuanye Biological Technology Co., Ltd. (Shanghai, China).The biological agents used in this study were shown in our previous report [36].

### 2.2. Expression of RpsL12–Rluc Fusion Protein

The genes of *Escherichia coli* RpsL12 (GenBank ID: CAD6001255.1) and renilla luciferase (GenBank ID: AAO48589.1) were assembled by using overlapping extensionpolymerase chain reaction to obtain the fused gene RpsL12–Rluc, which contained the gene of an amino acid linker (GSTSGSGKPGSGEGSTSG) and two restriction sites (EcoRI and XhoI). The RpsL12–Rluc gene was inserted into pET32a to construct the express vector RpsL12–Rluc–pET32a. These experiments were conducted bySangon Biotech Co., Ltd. (Shanghai, China). Then, the constructed recombinant plasmids were transformed into *Escherichia coli* BL21 (DE3) for culturation. After agarose gel electrophoresis analysis and DNA sequencing, the positive colonies were cultured in LB liquid culture media to express the fusion, and the expressionand characterization of the target protein were conducted followingthe procedures described in our previous report [36].

### 2.3. Recognition Mechanisms for AGs

The gene sequence of the *Escherichia coli* RpsL12 was translated into its amino acid sequence by using DNAman software. Then, its homological conformation was retrieved in PDB, and the optimal 3D model showing 100% homology with the present RpsL12 (PDB ID: 7P7S) was used for molecular docking. The intermolecular interaction mechanisms with the 7 drugs were determined by using YASARA 16.2.18, and the molecular docking wasconducted as described in our previous report [37].

### 2.4. Surface Plasmon Resonance (SPR)

For evaluation of its affinity, we used the surface plasmon resonance technique (SPR) to determine the bindingparameters of the *Escherichia coli* RpsL12 for the sevenAGs, including Ka, Kd, KD (Kd/Ka), and absolute affinity constant KA (Log2(KD)). In this study, the RpsL12–Rluc fusion protein was used to perform the SPR test directly, and the experiments were conducted according to our recently reported procedures [37]. The detailed SPR processes and the raw data areshown in the Supplementary Material (Figures S1–S7).

### 2.5. Preparation of Coating Conjugate

In this study, STR was coupled to BSA to prepare the coating conjugate STR–BSA by using the glutaraldehyde method, and the procedures were according to a previous report [8]. Briefly, 51 mg STR and 45 mg BSA were dissolved in 10 mL carbonate buffer (0.1 M, pH 9.5), and then 0.5 mL 0.1% glutaraldehyde was added. The mixture was stirred gently at room temperature for 6 h, and the obtained solution was dialyzed against water for 72 h to obtain the STR–BSA.

### 2.6. Development of the Bioluminescent Method

**Figure 2:**
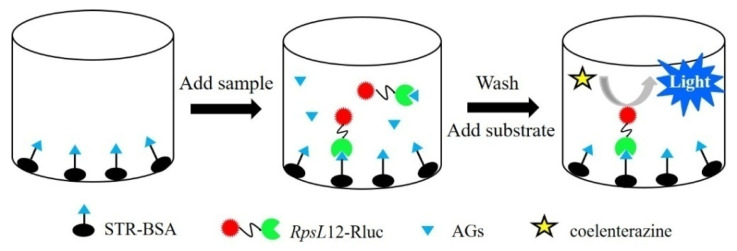
Schematic representation of the RpsL12–Rluc-fusion-protein-based bioluminescent method.

The indirect competitive bioluminescent method was performed as the schematic representation shown in Figure 2. Briefly, the coating conjugate STR–BSA was diluted with carbonate buffer (0.1 M, pH 9.5), and the solution was added into the wells of a white opaque 96-well microplate (100 μL/well) to be incubated at 4 °C overnight. After washing with water three times, the wells were blocked with 1% fetal calf serum (150 μL/well, 37 °C, 30 min). After washing, the solutions of 50 μL RpsL12–Rluc and 50 μL AGs (or sample extract) were added into the wells, and the plate was incubated at 37 °C for 30 min. After washing, 100 μL of coelenterazine-h solution was added, and the luminescence intensity (LI) of each well at 470 nm was measured. During the experiments, the concentration of STR–BSA, the concentration of RpsL12–Rluc, and the incubation time were optimized. Then, the sevenAGs were tested by the method, and the related IC_50_(half of inhibition concentration) and IC_10_(limit of detection)were determined.

### 2.7. Method Application

The residues of AGs in pork sample were extracted followingthe procedures described in a previous report [6]. Briefly, the homogenized pork sample (2 g) and the extraction solvent (3% trichloroacetic acid, 5 mL) were added into a centrifuge tube to be stirred vigorously for 5 min. Then, the tube was centrifuged at 3354× *g* for 5 min, and the solution was decanted into a clean tube. The pH of the solution was adjusted to 7.0 with 30% NaOH solution, and 50 μL of the solution was transferred into the microplate well for analysis. In this study, some pork samples obtained at the authorized slaughterhouses were used as blank samples to evaluate the method accuracy (intra-day and inter-day recovery) and precision (coefficient of variation, CV). Finally, 60 real pork samples purchased at several supermarkets in China were extracted and assayed by the present method.

## 3. Results and Discussions

### 3.1. Characterization of the RpsL12–Rluc Fusion Protein

**Figure 3:**
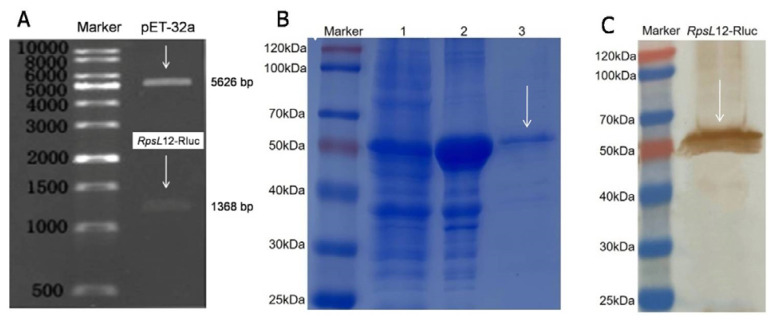
Results of (**A**) agarose gel electrophoresis, (**B**) SDS-PAGE (lane 1, supernatant; lane 2, inclusion body; lane 3, purified fusion protein), and (**C**) Western blotting for characterization of RpsL12–Rluc fusion.

In the previous reports, luciferase wasusually fused with an antibody to develop the bioluminescent immunoassay [29,30,31,32,33,34,35]. The present study, for the first time, produced a luciferase-fused receptor RpsL12–Rluc. The genes of RpsL12, Rluc, and linker were 372 bp, 942 bp, and 54 bp, respectively (a total of 1368 bp). The results showed that the expected RpsL12–Rluc gene of about 1368 bp was obtained (Figure 3A). The theoreticalmolecular weights of RpsL12 and Rluc were 13.74 kDa and 36.35 kDa, respectively. The results showed that the obtained RpsL12–Rluc was about 52.37 kDa (including His tag and the linker, 2.3 kDa, Figure 3B), which corresponded to the theoretic calculation. Furthermore, the RpsL12–Rluc was expressed as a soluble protein and inclusion body (Figure 3B), and for convenience, the soluble protein was purified for further study. As shown in Figure 3C, the Western blotting experiment showed that the target protein was obtained. On the basis of the above results, it could be said that the RpsL12–Rluc fusion protein was expressed.

### 3.2. Recognition Mechanisms for AGs

**Figure 4:**
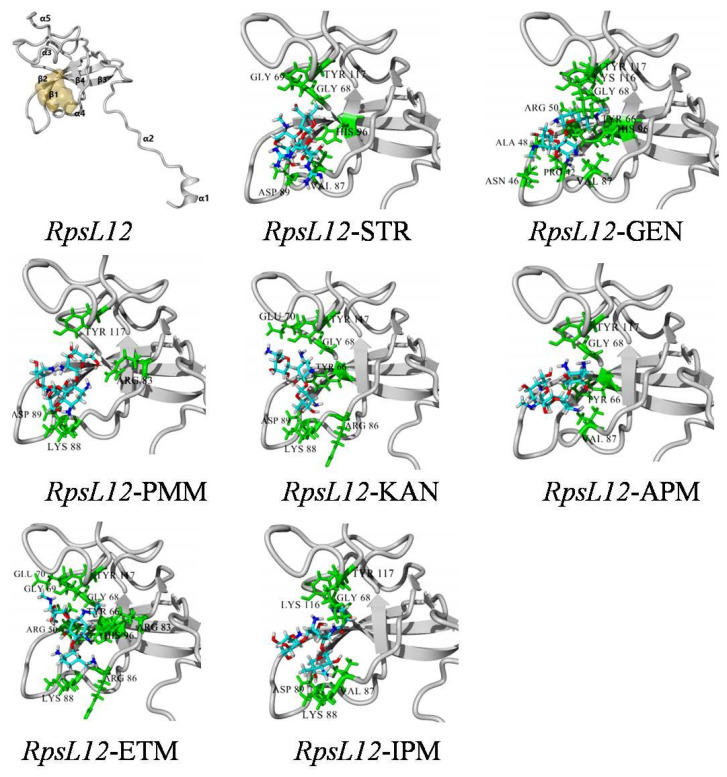
Homological model of the *E. coli* RpsL12 and its binding complexes with AGs.

In a previous report, the recognition mechanism of *E. coli* RpsL12 (PDB ID: 1VS5) for AGs (streptomycin) was studied, but the recognition mechanisms for other AGs were omitted [25]. In the present study, the optimal model showing 100% homology with the present *E. coli* RpsL12 (PDB ID: 7P7S) was used for molecular docking. As shown in Figure 4, the present *E. coli* RpsL12 was composed of fourβ-sheets and five α-helixes, and the α4, α5, β2, β3, and β4 constructed the tunnel-like pocket. Then, the sevenAGs were docked with the model to study their intermolecular interactions.

As shown in Table 1, hydrophobic interaction involved in the bindings with all of the sevenAGs, and hydrogen bond was involved in the bindings with sixAGs. Among the contact amino acids, TYR117 from α5 interacted with sevendrugs, and GLY68 from β3 interacted with sixdrugs. As can be seen from the docking complexes shown in Figure 4, GLY68 and TYR117 constructed the bottom of the binding pocket. As shown in Table 1, STR, IPM, APM, GEN, ETM, and KAN simultaneously interacted with the two amino acids, and PMM only interacted with TYR117. Therefore, the two amino acids were the key contact amino acids.

As shown in Figure 1, the sevenAGs were composed of threeor four rings, and their common core ring was 2-deoxystreptamine, except for STR, whose core ring was streptidine. The binding sites for the sevendrugs were mainly on the substituted rings and the side chains, and thoseon their core rings were less (Figure 1). Because the general molecular structures of the sevendrugs were similar and they could all contact with the pocket bottom, the RpsL12 showed similar binding energies for them (5.44–6.32 kcal/moL, Table 1). This meant the recognition abilities of the RpsL12 for the sevenAGs were comparable.

In this study, the binding affinities for the sevenAGs were also determined (Ka, Kd, KD, and KA). The results showed that the RpsL12 showed similar KA for sixAGs (20.515–25.235), except for ETM (12.502, Table 1). This meant the affinities of the RpsL12 for the sevenAGs were comparable, identical with the result of molecular docking. This work for the first time thoroughly studied the intermolecular interactions of E. coli RpsL12 with AGs, and the obtained recognition mechanisms were more comprehensive and detailed than the previous results [25].

### 3.3. Characterization of the Coating Conjugate

**Figure 5:**
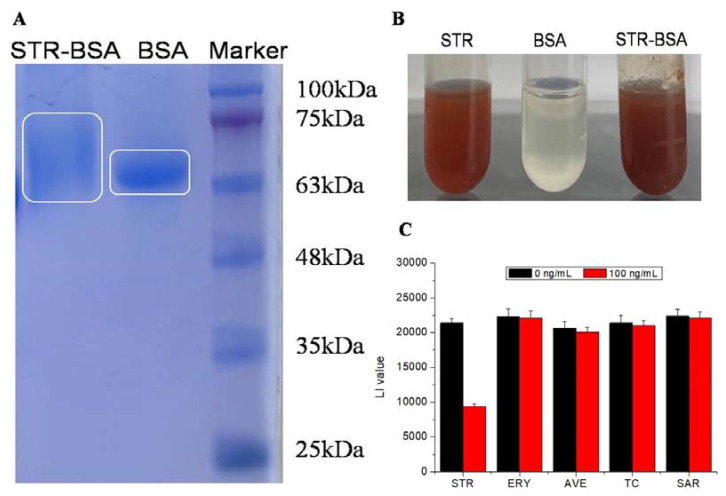
Characterization of the coating conjugate STR–BSA by using (**A**) SDS-PAGE and (**B**) maltol reaction. (**C**) LI values when testing STR and other drugs (SAR =sarafloxacin, TC = tetracycline, AVE = avermectin, ERY = erythromycin, 0 and 100 ng/mL; STR–BSA 1:1000, RpsL12–Rluc 1:1000).

For development of an indirect competitive method, STR was coupled with BSA to prepare the coating conjugate. As shown in Figure 5A, the SDS-PAGE result showed that the molecular weight of STR-BSA was larger than that of BSA, indicating that some STR molecules were linked with BSA. Furthermore, the streptose structure inthe STR molecule has aunique maltol reaction, and thusBSA, STR, and STR–BSA were all characterized by the special assay. As shown in Figure 5B, STR and STR–BSA provided a positive result, but BSA did not providethe expected result. These results proved that the conjugate STR–BSA was obtained.

For the evaluation of whetherSTR–BSA could combine with RpsL12–Rluc to develop an indirect competitive method, some experiments were conducted. During the experiments, the microplate wells were coated with the STR–BSA, and then the RpsL12–Rluc was mixed with STR and four other classes of drugs (erythromycin, avermectin, tetracycline, sarafloxacin) to perform the competition. As shown in Figure 5C, the luminescence intensities (LI) of all the wells were high when the drug concentration was 0 ng/mL, indicating thatSTR–BSA could bind with RpsL12–Rluc. Furthermore, the LI value decreased dramatically when testing STR (100 ng/mL), and the inhibition ratio (1-LI/LI_0_) was 56%. This meant thatSTR–BSA and RpsL12–Rluc could be used to develop a competitive method for determination of AGs. However, the LI values when testing other drugs (100 ng/mL) decreased a little, and the inhibition ratios were all lower than 2% (Figure 5C). This meant that the *E. coli* RpsL12 only showed specific recognition for AGs, and the developed method should be able to determine AGs specifically, which was because the *E. coli* RpsL12 was the receptor of AGs but not the receptor of other drugs.

### 3.4. Optimization of Method Parameters

**Figure 6:**
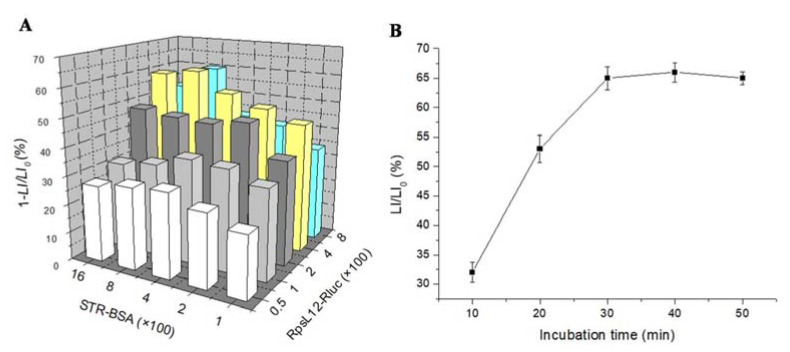
Results for the optimization of (**A**) the concentrations of RpsL12–Rluc and STR–BSA, and (**B**) incubation time (STR 100 ng/mL).

This study for the first time developed an RpsL12–Rluc-fusion-based bioluminescent method for the determination of AGs. In this study, STR was used to optimize severalassay parameters (STR–BSA concentration, RpsL12–Rluc concentration, and competition time). During the experiments, the microplate wells were coated with different dilutions of STR–BSA, and then STR (100 ng/mL) and different dilutions of RpsL12–Rluc were added into the wellsfor analysis. The results showed that the use of 1:800 of STR–BSA and 1:400 of RpsL12–Rluc achieved the highest inhibition ratio(Figure 6A), and thereforethe two dilutions were selected for the following experiments. In addition, the RpsL12–Rluc(1:400) and STR (100 ng/mL) were added into the STR–BSA (1:800)-coated microplate wells to be incubated for different times. Results showed that the competition reached equilibrium when the incubation time was increased to 30 min (Figure 6B), and thusthis competition time was selected for the following experiments.

### 3.5. Method Performances

**Figure 7:**
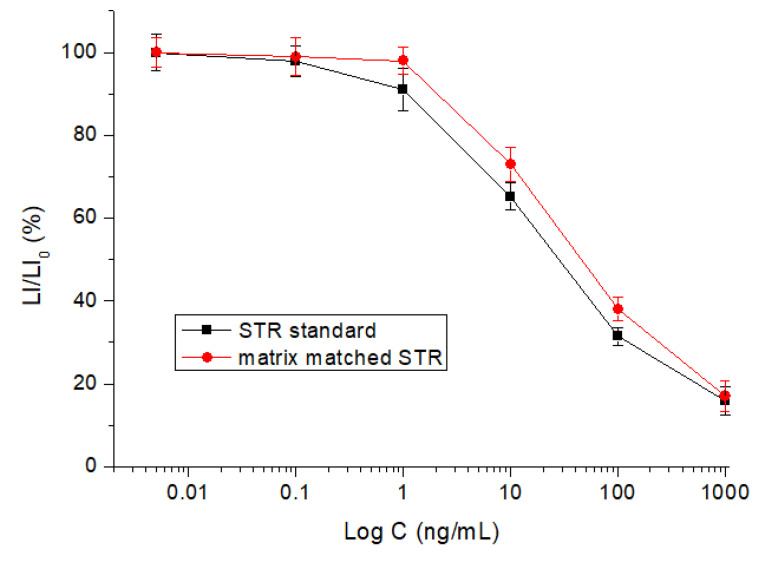
Competitive curves of STR standard and matrix-matched STR (0.01–1000 ng/mL).

**Figure 8:**
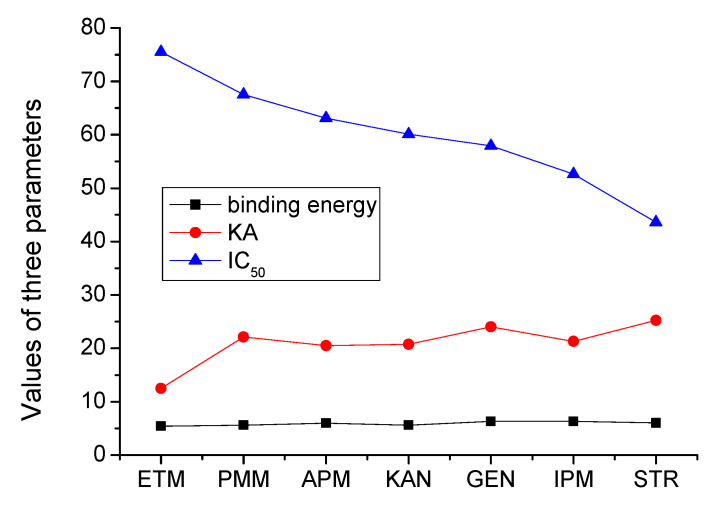
Integration of KA, IC_50_, and binding energy of the 7 AGs.

After optimization of the parameters, the sevenAGs were tested by the method. The results showed that the IC_50_ of the sevendrugs ranged from 43.6 to 75.5 ng/mL, and the limits of detection (IC_10_) ranged from 0.51 to 1.1 ng/mL (Table 1). The representative competitive inhibition curve of STR was shown in Figure 7. During preparation of the paper, the IC_50_, KA, and binding energyof the sevenAGs were integrated with the aim of a comprehensive understanding of their relationship. As shown in Figure 8, their binding energies were comparable, but the IC_50_ values were negatively correlated with the KA values. For example, the IC_50_ of ETM (75.5 ng/mL) was the highest and its KA (12.502) was the lowest, and the IC_50_ of STR (43.6 ng/mL) was the lowest and its KA (25.235) was the highest. This meant that a higher affinity represented a lower IC_50_, i.e., a higher sensitivity. These results revealed that the comparable binding energies for the sevenAGs provided the base to develop the broad specific bioluminescent method, whereas the sensitivity for a specific drug was determined by the binding affinity for it.

### 3.6. Sample Determination

In this study, the sevenAGs were spiked into the blank samples at levels of 10–600 ng/g for analysis. The intra-assay recoveries (73.8–96.2%), the inter-assay recoveries (77.5–94.8%), and the coefficients of variation (5.9–12.9%) areshown in Table 2. The matrix matched competitive curve of STR was generally similar to the STR standard (Figure 7), indicating the influence of sample matrix was minimum. Finally, the 60 real pork samples were determined. It was found that three real samples were determined as positive samples (7 ng/g, 20 ng/g, and 48 ng/g; expressed as STR), but the AGs species could not be identified. This meant that an instrumental method was required to confirm the positive results and determine the specific drug. However, such an instrumental method has not been reported thusfar. Still, the bioluminescent method reported in this study could be used as a rapid screening tool for multi-determination of the sevenAGs in a large number of meat samples.

### 3.7. Comparison with Related Immunoassays

During preparation of the paper, the previously reported enzyme-linked immunosorbent assays (ELISA) for AGs [5,6,7,9,10,11,14] were summarized in Table 3 to compare with the present method. First, this study for the first time used RpsL12–Rluc fusion as a recognition reagent for the detection of AGs, and the production of the fusion protein was simpler and more rapidthan the productions of those antibodies. Second, this study for the first time reported a bioluminescent method for detection of AGs. Third, the operation steps, detection spectrum, assay time, and sensitivity of the present method were better than or comparable to those methods. With general consideration of the above-mentioned points, the present method was superiorto those methods.

## 4. Conclusions

All of the previous immunoassays for determination of AGs used the conventional antibody as a recognition reagent. In this study, a novel fusion protein by linking *Escherichia coli* RpsL12 with renilla luciferase was generated. The receptor part in this fusion protein was able tosimultaneously recognizesevenAGs, and the developed indirect competitive bioluminescent methodwas able tosimultaneously determine the sevenAGs in pork. After comparison, the method performances (operation steps, detection spectrum, assay time, and sensitivity) were generally better than the previously reported ELISA methods for AGs. Therefore, the present bioluminescent method could be used as a practical tool for the routine screening of a low level of AG residues in meat samples.

## Figures and Tables

**Table 1:** Results of molecular docking, affinity, and sensitivity for the 7 AGs.

Drug	Binding Energy (kcal/mol)	Contact Amino Acid		Ka (1/Ms)	Kd (1/s)	Kd (1/s)	KA	IC_50_ (ng/mL)	LOD (ng/mL)
		Hydrogen Bond	Hydrophobic Interaction						
STR	6.02	GLY69	GLY68 VAL87 ASP89 HIS96 TYR117	2.05 × 10³	5.19 × 10^−5^	2.53 × 10^−8^	25.235	43.6	0.73
APM	5.99	TYR66	TYR66 GLY68 VAL87 TYR117	1.90 × 10^2^	1.27 × 10^−4^	6.67 × 10^−7^	20.515	63.1	0.86
GEN	6.29	--	PRO42 ASN46 ALA48 ARG50 TYR66 GLY68 VAL87 HIS96 LYS116 TYR117	4.46 × 10^2^	2.57 × 10^−5^	5.77 × 10^−8^	24.048	57.9	0.68
KAN	5.6	TYR66 GLU70 ARG86 LYS88 TYR117	GLY68 GLU70 LYS88 ASP89 TYR117	4.98 × 10^2^	2.83 × 10^−4^	5.69 × 10^−7^	20.746	60.1	0.84
ETM	5.44	HIS96 TYR117	ARG50 TYR66 GLY68 GLY69 GLU70 ARG83 ARG86 LYS88 HIS96 TYR117	8.82 × 10^1^	1.52 × 10^−2^	1.72 × 10^−4^	12.502	75.5	1.1
IPM	6.32	LYS116	GLY68 VAL87 LYS88 ASP89 TYR117	4.37 × 10³	1.70 × 10^−3^	3.90 × 10^−7^	21.290	52.6	0.51
PMM	5.61	ARG83 ASP89	LYS88 TYR117	4.61 × 10^2^	9.97 × 10^−5^	2.16 × 10^−7^	22.140	67.5	0.82

**Table 2:** Recoveries from AG-fortified blank pork samples (*n* =6).

Analyte	Fortified (ng/g)	Intra-Day		Inter-Day		Analyte	Fortified (ng/g)	Intra-Day		Inter-Day	
		Recovery (%)	CV (%)	Recovery (%)	CV (%)			Recovery (%)	CV (%)	Recovery (%)	CV (%)
STR	10	78.4	5.9	82.7	9.6	IPM	10	80.5	6.8	77.5	8.9
	100	87.5	8.8	89.9	11.5		50	87.4	8.7	81.7	9.9
	600	88.3	7.7	82.0	9.0		100	83.2	8.1	94.3	9.3
APM	10	89.5	7.8	90.4	8.9	PMM	10	89.1	7.7	85.7	8.8
	50	84.6	8.5	94.2	8.4		50	96.2	7.4	89.1	11.7
	100	93.8	6.3	91.8	10.7		100	91.5	8.6	82.4	12.9
GEN	10	79.4	9.4	78.5	9.5	ETM	10	73.8	8.3	91.9	10.2
	50	83.8	8.3	86.7	8.9		50	95.7	7.9	94.8	8.6
	100	88.4	8.8	83.7	8.7		100	91.6	7.2	90.7	8.0
KAN	10	80.6	7.4	93.7	12.8						
	50	84.8	9.1	92.7	10.7						
	100	92.7	9.4	88.6	8.4						

**Table 3:** The main results of previous enzyme-linked immunosorbent assays (ELISAs) for AGs.

Recognition Element	Immunoassay	Detection Spectrum	Test Time (after Add Sample)	Limit of Detection(ng/g)	Ref.
Streptomycin pAb	ELISA	1 drug	30 min	1	[5]
Kanamycin mAb	ELISA	2 AGs	30 min	0.9–1.8	[6]
Gentamycin pAb	ELISA	1 drug	2 h	14.16	[7]
Kanamycin mAb	ELISA	2 AGs	100 min	0.022	[9]
Streptomycin mAb	ELISA	2 AGs	40 min	0.09–1.37	[10]
Streptomycin mAb	fluorescence ELISA	1 drug	60 min	0.005	[11]
Commercial Ab	chemiluminescent ELISA	1 drug	60 min	9.4	[14]
*E. coli* RpsL12	bioluminescent method	7 AGs	30 min	0.51–1.1	This study

## Data Availability

The data presented in this study are available on request from thecorresponding author.

## Supplementary Materials

The following supporting information can be downloaded at: https://www.mdpi.com/article/10.3390/foods12020284/s1, Figure S1: Binding kinetics of apramycin with different concentrations of RpsL12-Rluc; Figure S2: Binding kinetics of gentamicin with different concentrations of RpsL12-Rluc; Figure S3: Binding kinetics of streptomycin with different concentrations of RpsL12-Rluc; Figure S4: Binding kinetics of paromomycin with different concentrations of RpsL12-Rluc; Figure S5: Binding kinetics of etimicin with different concentrations of RpsL12-Rluc; Figure S6: Binding kinetics of kanamycin with different concentrations of RpsL12-Rluc; Figure S7: Binding kinetics of isepamicin with different concentrations of RpsL12-Rluc.
