# Supplementary material for: Production of Ribosomal Protein S12/Renilla Luciferase Fusion and Development of a Bioluminescent Method for Detection of Aminoglycosides in Pork and Studying Its Recognition Mechanism
Source: Foods. 2023 Jan 7;12(2):284. doi: 10.3390/foods12020284 (PMC9858597; doi:10.3390/foods12020284)
Supplement: Supplementary file 1 [file foods-12-00284-s001.zip › foods-2109983-supplementary.pdf]

SUPPLEMENTARY MATERIALS FOR

Production of ribosomal protein S12/renilla luciferase fusion and development of a bioluminescent method for detection of aminoglycosides in pork and studying its recognition mechanism

Wan Qiu Xia [1], Lei Zhang [1], Jian Ping Wang [1,2]

[1] College of Veterinary Medicine, Hebei Agricultural University, Baoding Hebei, 071000, China

[2] Veterinary Biological Technology Innovation Center of Hebei Province, Baoding Hebei 071000, China

Surface plasmon resonance experiments and raw data

The 7 AGs were diluted with DMSO, and these solutions were printed on a Photo-Cross-Linker SensorCHIP™ by using Biodot™ AD1520 Array Printer respectively. At the same time, rapamycin as the positive control and DMSO as the negative control were also printed on the chip. The chip was dried and subjected to photocross-linking treatment under UV irradiation. Then the chip was inserted into the Berthold bScreen LB 991 Microarray System for use. The RpsL12-Rluc fusion protein at concentrations of 10, 40, 160, 640 and 2560 nM were injected into the Berthold bScreen LB 991 Microarray System for analysis respectively. The operation conditions were: flow rate 0.5 μL/s, binding time 600 s, dissociation time 360 s, and temperature 4 °C.

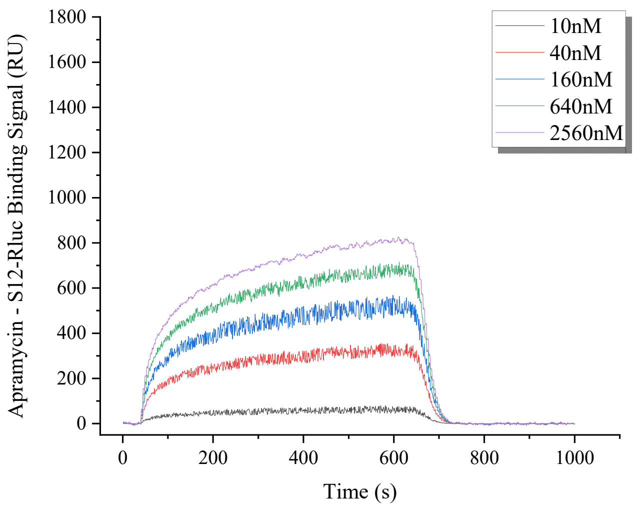

Figure S1. Binding kinetics of apramycin with different concentrations of RpsL12-Rluc.

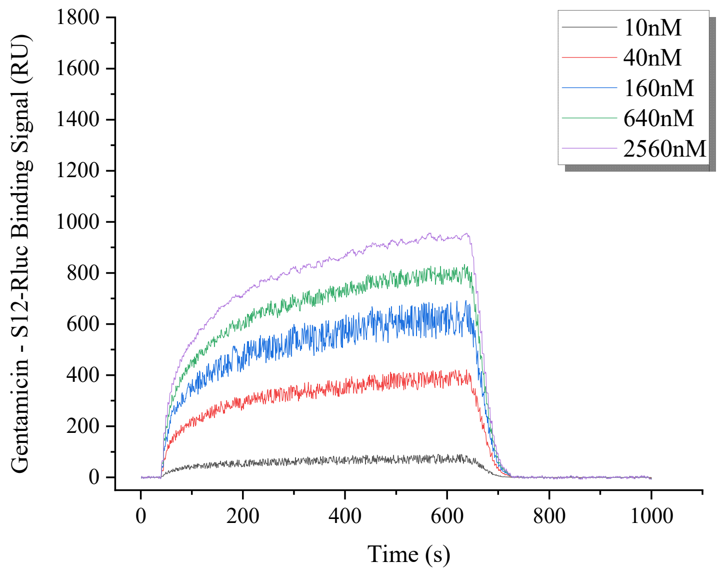

Figure S2. Binding kinetics of gentamicin with different concentrations of RpsL12-Rluc.

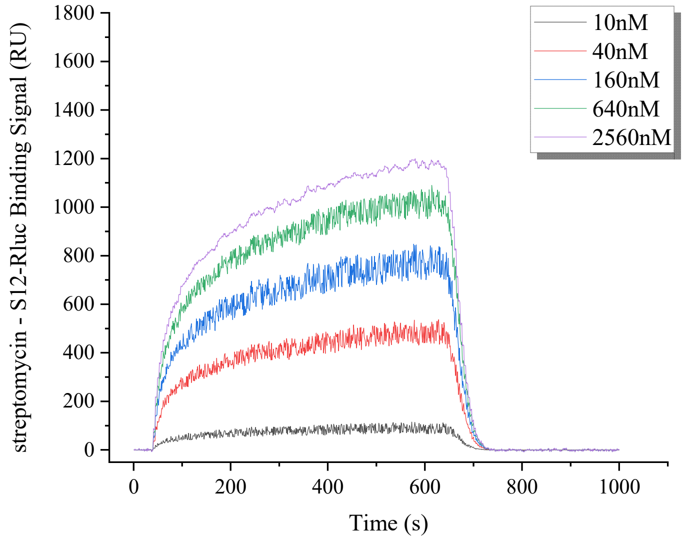

Figure S3. Binding kinetics of streptomycin with different concentrations of RpsL12-Rluc.

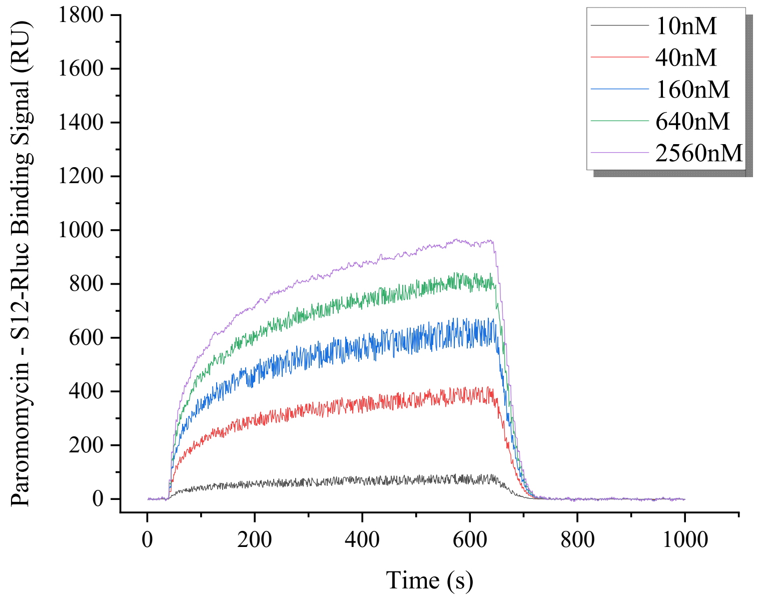

Figure S4. Binding kinetics of paromomycin with different concentrations of RpsL12-Rluc.

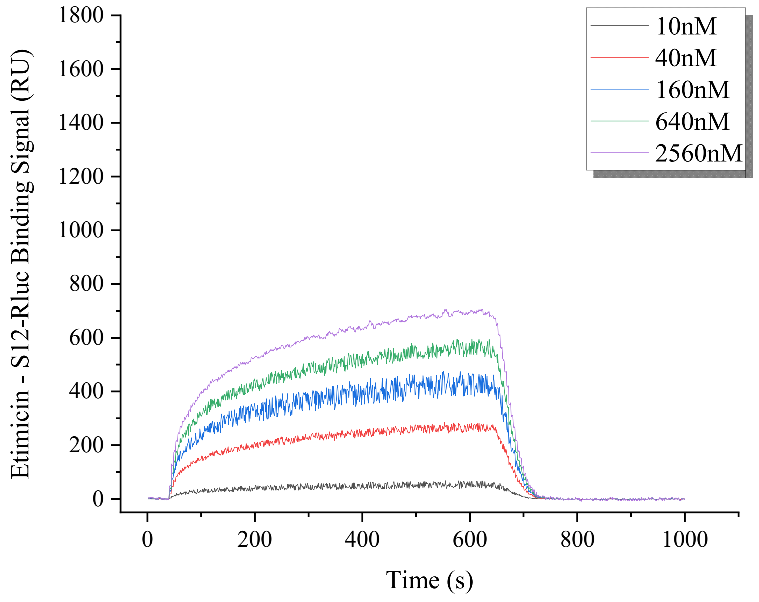

Figure S5. Binding kinetics of etimicin with different concentrations of RpsL12-Rluc.

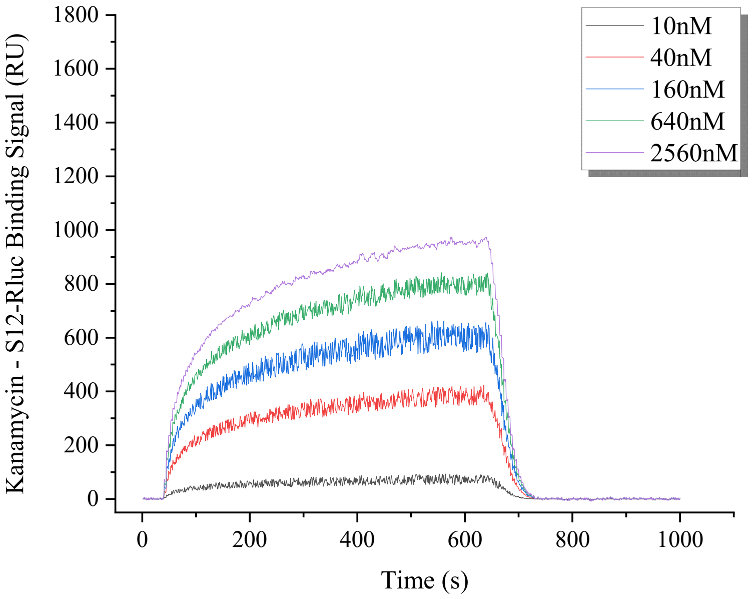

Figure S6. Binding kinetics of kanamycin with different concentrations of RpsL12-Rluc.

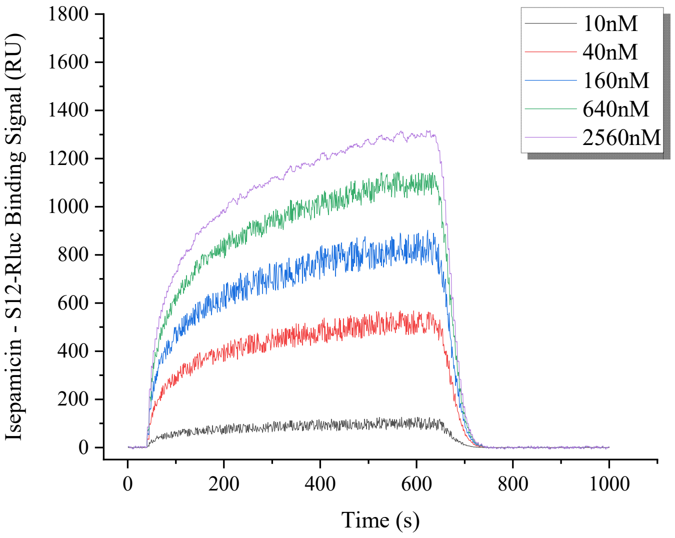

Figure S7. Binding kinetics of isepamicin with different concentrations of RpsL12-Rluc.
